# Bioinformatics and machine learning-driven discovery of candidate tissue diagnostic markers for endometriosis with experimental verification

**DOI:** 10.3389/fendo.2026.1802649

**Published:** 2026-05-22

**Authors:** Juan Du, Shanshan Zhao, Qiuju Feng, Weiping Cheng

**Affiliations:** 1 The First Affiliated Hospital of Heilongjiang University of Chinese Medicine, Harbin, Heilongjiang, China; 2 Heilongjiang University of Chinese Medicine, Harbin, Heilongjiang, China; 3 Gynecology Department 1, The Second Affiliated Hospital of Heilongjiang University of Chinese Medicine, Harbin, Heilongjiang, China; 4 Integrated Traditional Chinese and Western Medicine Rehabilitation Medical Center, Heilongjiang Provincial Hospital, Harbin, Heilongjiang, China; 5 Acupuncture and moxibustion Ward 5, The Second Affiliated Hospital of Heilongjiang University of Traditional Chinese Medicine, Harbin, Heilongjiang, China; 6 Department of Acupuncture and Moxibustion, The First Affiliated Hospital of Heilongjiang University of Chinese Medicine, Harbin, Heilongjiang, China

**Keywords:** bioinformatics, biomarkers, endometriosis, machine learning, pathogenic mechanism

## Abstract

**Background:**

Endometriosis is a complex gynecological disorder lacking reliable biomarkers. This study aimed to identify core diagnostic genes through integrated computational approaches. Multiple endometriosis transcriptomic datasets were analyzed.

**Methods:**

Differential expression analysis and Weighted Gene Co-expression Network Analysis (WGCNA) screened disease-associated genes. Functional enrichment, Protein-Protein Interaction (PPI) network construction, and an ensemble machine learning framework (113 algorithm combinations) were employed to refine hub genes. Diagnostic performance was validated via ROC analysis. Immune infiltration was characterized using CIBERSORT.

**Results:**

Four genes (COL6A3, BGN, LAMA4, THBS2) were identified as robust candidate tissue diagnostic markers, showing consistent upregulation and high discriminatory power (AUC > 0.80). They are implicated in extracellular matrix remodeling. Immune dysregulation was observed, featuring elevated M1 macrophages and plasma cells, alongside reduced resting NK cells, with hub genes correlating with specific immune subsets. To functionally validate these findings, a mice endometriosis model was established and exhibited histopathological features consistent with the disease, including ectopic lesion formation and altered glandular architecture. qPCR and Western blot analyses confirmed significant upregulation of COL6A3, BGN, LAMA4, and THBS2 at both transcriptional and protein levels in ectopic endometrium, further supporting their role in disease pathogenesis.

**Conclusion:**

COL6A3, BGN, LAMA4, and THBS2 represent promising candidate tissue diagnostic markers for endometriosis, linked to extracellular matrix and immune microenvironment alterations, providing novel insights for future research and clinical translation.

## Introduction

Endometriosis represents a chronic inflammatory condition influenced by estrogen, significantly impacting the health and quality of life for women in their reproductive years (1). This condition is marked by the presence of tissue resembling the endometrium growing beyond the uterine cavity, primarily involving pelvic organs but potentially affecting multiple sites throughout the body. Similar to inflammatory bowel disease, endometriosis exhibits a typical “relapse-remission” disease course pattern. Under hormonal regulation, the lesions undergo cyclic bleeding, inflammatory responses, and tissue repair, leading to progressive fibrosis, adhesion formation, and nerve infiltration (2, 3). These processes ultimately result in complex clinical symptoms such as chronic pelvic pain and infertility (4). Among the multifactorial pathogenesis of Endometriosis, genetic susceptibility has been confirmed to play a critical role (5). Despite a global prevalence as high as 10%, the exact pathogenic genes and molecular mechanisms of endometriosis remain incompletely understood. Current treatment strategies primarily focus on symptom control and disease delay but are associated with significant side effects and high recurrence rates (6, 7).

This situation underscores the necessity of in-depth exploration of the key molecular features of endometriosis, which would provide important clues for creating new diagnostic biomarkers and focused treatment strategies. Advances in high-throughput sequencing have facilitated genome-wide association studies (GWAS) in identifying multiple genetic loci associated with endometriosis risk (8). To date, a substantial body of research has been dedicated to identifying core genes involved in endometriosis. Among these, ESR1 and PGR are the most consistently validated and widely recognized key genes, reflecting the central role of estrogen signaling in disease pathogenesis (9). In addition, other candidate genes have been identified through transcriptomic and proteomic approaches. For instance, BGN (biglycan) has been characterized as a novel estrogen-related gene associated with ovarian endometriosis and has also been identified as a key biomarker for the malignant progression from endometriosis to endometrial cancer (10, 11). Furthermore, recent studies have identified THBS2, CSF1R, IL-4R, and FLT1 as potential biomarkers, further expanding the molecular landscape of the disease (12). Beyond protein-coding genes, emerging evidence highlights the clinical potential of exosomal microRNAs as diagnostic biomarkers for endometriosis (13). Despite these advances, the number of well-established core genes remains limited, and the genetic architecture of endometriosis is far from complete. Given the high heterogeneity and complex pathogenesis of the disease, it is likely that additional critical genes remain to be fully characterized. Therefore, continued efforts to discover and validate novel key genes are essential for improving early screening, diagnostic accuracy, and the development of targeted therapeutic strategies for endometriosis.

To systematically decipher the transcriptomic characteristics of endometriosis and identify core pathogenic genes, this study adopted an integrated bioinformatics analysis strategy. Multiple transcriptome datasets from endometriosis patients and health controls were obtained from the Gene Expression Omnibus (GEO) database. The initial dataset served as the main training set for conducting in-depth analysis, the subsequent two datasets served as independent test sets for validation, and the final dataset was utilized for supplementary verification, ensuring the reliability of the research findings. The study first identified significant differentially expressed genes (DEGs) through a comparison of gene expression patterns between endometriosis patients and control subjects. Based on these DEGs, Weighted Gene Co-expression Network Analysis (WGCNA) was employed to construct gene modules and identify key gene sets most relevant to the pathological processes of endometriosis. Subsequently, GO functional annotation and KEGG pathway enrichment analyses were performed on the screened key genes to elucidate the potential molecular mechanisms of endometriosis at the functional level. To further refine the core genes, the study integrated Protein-Protein Interaction (PPI) network analysis and machine learning methods to screen for hub genes with the strongest predictive power. These genes showed strong diagnostic capabilities in both the training and test datasets. The research additionally assessed how these genes were expressed in various endometriosis tissues and explored their links to clinical parameters. The CIBERSORT algorithm was employed to analyze immune cell infiltration patterns in endometriosis tissues, focusing on identifying key immune cell types with abnormal changes while exploring the interactions between hub genes and the immune microenvironment.

## Methods

### Key gene screening research design

**Figure 1:**
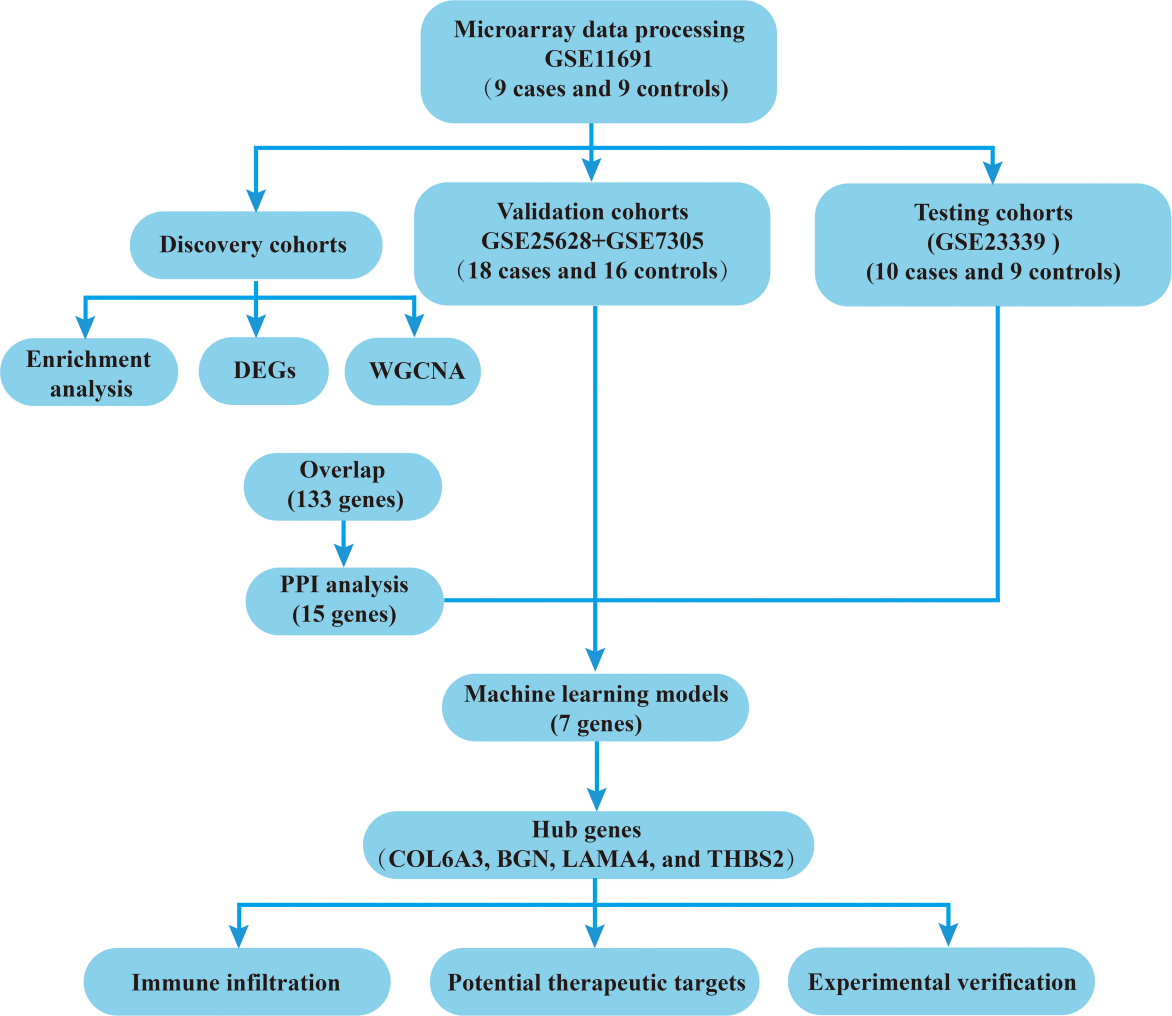
Technical flowchart of the present research.

Using the GEO database, this study identified overlapping genes associated with endometriosis by applying WGCNA and DEG analysis. Next, key hub genes were further selected through PPI network analysis combined with machine learning techniques. The full analytical process is depicted in **Figure 1**.

### Data collection and preprocessing

The study utilized the EM related training dataset GSE11691 from the GEO database (9 cases of EM and 9 controls). Two additional datasets, GSE25628 (8 EM cases and 6 controls) and GSE7305 (10 EM cases and 10 controls), were combined to form the test set. Prior to merging, batch effects between GSE25628 and GSE7305 were removed using the “sva” R package (version 3.50.0). The merged dataset served as the test group. Additionally, GSE23339 (10 EM cases and 9 controls) was used as an independent external validation set. All expression data were log−transformed and normalized using the “normalizeBetweenArrays” function from the limma R package (v3.58.1) (14).

### Analysis of DEGs through a weighted gene co-expression network approach

For WGCNA development targeting DEGs, the “WGCNA” R package (version 1.73) was employed to pinpoint gene modules closely linked to endometriosis (15). Initially, our investigation focused on soft threshold powers (β) to create a scale-free network topology. Following this, we created a weighted adjacent matrix, subsequently transformed into a topological overlap matrix (TOM). In the case of hierarchical clustering, a measure of dissimilarity derived from TOM was computed. Utilizing the dynamic tree cutting technique, we pinpointed unique modules by grouping genes according to their similarity in expression. Genes exhibiting a significant correlation with clinical characteristics of endometriosis were chosen for further examination. We acknowledge that using DEGs as the input for WGCNA may introduce potential bias in network topology, as pre-filtering removes genes with low fold-change but high co-expression variation. However, this approach was employed as a conservative initial screening step to prioritize genes that are both differentially expressed and co-expressed in disease-relevant modules. Importantly, the robustness of our core gene candidates was subsequently validated through orthogonal approaches, including PPI network analysis, ensemble machine learning (113 algorithm combinations), external dataset validation, and experimental verification, which collectively support the biological significance of the identified genes independent of the initial WGCNA input.

### Examining the enrichment of GO and KEGG in key genes

To investigate the biological functions and signaling pathways of endometriosis-associated genes, the overlapping genes identified from weighted gene co-expression network analysis (WGCNA) and differentially expressed genes (DEGs) were subjected to enrichment analysis. Gene Ontology (GO) and Kyoto Encyclopedia of Genes and Genomes (KEGG) enrichment analyses were performed using the R packages “clusterProfiler” and “DOSE” (v3.28.2) (16, 17). This approach facilitates the annotation of genes in terms of human diseases, biological processes, and signaling pathways, which may reveal their roles in the pathogenesis and progression of endometriosis (18).

### Construction and topological analysis of a PPI network

A protein−protein interaction (PPI) network was generated from the overlapping genes of the DEG set and the WGCNA results using the STRING database, and subsequently visualized with Cytoscape (version 3.9.1) (19, 20). To identify high-priority hub genes, the top 20 nodes based on their connectivity scores were determined using all five topological analysis algorithms integrated within the cytoHubba Cytoscape plug-in. Additionally, the DEGREE algorithm was applied to further refine the most connected genes within the network (21).

### Ensemble machine learning for feature selection

Five crucial genes pinpointed through PPI network analysis were later integrated into various machine learning models (22). Twelve algorithms were employed, including regularization models (Lasso, Ridge, ElasticNet), generalized linear approaches (StepGLM, GLMBoost, PLSRGLM), ensemble methods (Random Forest, GBM, XGBoost), pattern recognition classifiers (SVM, LDA, Naïve Bayes), and gradient−based boosting techniques. The design, embracing multiple paradigms, incorporated linear regression along with sophisticated nonlinear structures to guarantee extensive analytical strength.

To ensure robustness, the computational framework implemented a dual-stage strategy: (1) feature ranking and predictor screening via recursive feature elimination, followed by (2) predictive modeling using stacked generalization. The process was embedded within a stratified 10-fold cross-validation framework, generating 113 unique model configurations through systematic hyperparameter optimization. Subsequently, the analysis proceeded with four algorithms capable of integrated feature selection: Lasso, Random Forest, StepGLM, and GLMBoost. These methods were critical in further refining gene selection, effectively reducing the candidate pool to a more manageable subset.

Following the preliminary filtering phase, eight more algorithms were employed to adapt predictive models, utilizing the gene sets chosen by the preceding four algorithms. The stringent method led to the examination of 113 different model pairings, each subject to hyperparameter adjustments and adjustments within the strict parameters of a 10-fold cross-validation structure. Following this, every created model combination underwent assessment through both training and testing groups. The effectiveness of the model was evaluated through the computation of area under the curve (AUC) scores, key indicators for assessing the model’s efficiency. The model that showed the greatest average AUC score among both training and testing groups was considered the best. The extraction of trait genes from this highly effective model yielded crucial understanding of the genetic elements affecting traits of endometriosis.

### Diagnostic efficacy, expression profiling, and correlative patterns of hub genes

A receiver operating characteristic (ROC) analysis was conducted to evaluate the diagnostic effectiveness of potential feature genes in endometriosis cases. The area under the curve (AUC) for each gene was computed with the “pROC” package (v1.18.5) in R, measuring its ability to discriminate between endometriosis and control samples (23). Genes achieving an AUC exceeding 0.7 in both the training and validation sets were retained as promising diagnostic biomarkers. Next, a logistic regression model was built via the “glmnet” R package (v4.1-8) to evaluate the aggregate discriminative power of the selected genes. Finally, inter-gene correlations within the validation cohort were analyzed and visualized using the “PerformanceAnalytics” package (v2.0.8).

### Assessment of immune cell infiltration via CIBERSORT and correlation analysis with hub genes

The immune landscape within endometriosis was characterized by using CIBERSORT, an analytical tool that applies linear support vector regression to deconvolute bulk tissue transcriptomic data and infer relative abundances of specific immune cell types. The reference dataset provided by CIBERSORT (https://cibersortx.stanford.edu/) enabled the quantification of 22 distinct immune cell phenotypes. The LM22 signature matrix (containing 22 distinct immune cell subtypes, including naive B cells, memory B cells, plasma cells, CD8+ T cells, CD4+ naive T cells, CD4+ memory resting T cells, CD4+ memory activated T cells, follicular helper T cells, regulatory T cells, gamma delta T cells, resting NK cells, activated NK cells, monocytes, M0 macrophages, M1 macrophages, M2 macrophages, resting dendritic cells, activated dendritic cells, resting mast cells, activated mast cells, eosinophils, and neutrophils) was used as the reference. CIBERSORT was run with 1,000 permutations to compute p-values for the deconvolution results. Samples with p < 0.05 after deconvolution were retained for downstream analysis, ensuring the reliability of immune infiltration estimates.

Subsequent correlation analysis between the identified hub genes and key immune infiltrates revealed significant alterations in the endometriosis microenvironment compared to controls. R programming (version 4.3.3) was utilized for performing statistical analyses. Pearson correlation coefficients were utilized to evaluate the patterns of hub gene expression. Additionally, Spearman’s rank-order technique was utilized to assess possible links between the infiltration patterns of immune cells and the transcriptional activity of pinpointed central genes. Within this study, a p-value less than 0.05 was considered to hold statistical significance.

### Establishment of the mice EMs model

Twelve female C57BL/6 mice (22 ± 5g) were used in this study. All recipient mice were confirmed to be in the diestrus stage of the estrous cycle by vaginal cytology prior to surgery to minimize cycle-related variability in endometrial implantation. Animals were randomly assigned to either a normal control group or an endometriosis model group (n = 6 per group) using a random number table. The autologous transplantation model used in this study does not require exogenous estrogen supplementation, as the recipient’s own ovaries provide endogenous estrogen to support ectopic lesion growth. To establish the endometriosis model, uterine horns were aseptically excised, longitudinally incised, and sectioned into approximately 2×2 mm explants in sterile phosphate-buffered saline (PBS). Recipient mice were anesthetized, and a midline laparotomy carried out to access the abdominal cavity. One uterine tissue explant was surgically sutured onto either the abdominal wall or a vascularized region of the mesentery. The abdominal incision was then closed, and the animals were individually housed for postoperative recovery. After 3–4 weeks of *in vivo* growth, well-vascularized ectopic lesions were macroscopically observed at the implantation sites. Successful model induction was confirmed by histopathological analysis, wherein hematoxylin and eosin (HE) staining identified the presence of characteristic endometrial glands and stroma within the ectopic implants. All experimental procedures followed the ethical guidelines of Heilongjiang University of Chinese Medicine and received approval from the Institutional Animal Care and Use Committee (IACUC).

### H&E staining

Endometrial tissue samples (2 cm²) were fixed in 4% paraformaldehyde at 4 C for 12 hours, followed by a running water rinse. The tissues underwent two rounds of impregnation with melted paraffin at 58–60 C, each lasting 2 hours, before being embedded in paraffin blocks. Using a microtome, sections were continuously cut at 4 μm thickness and then baked for 2 hours at 60 C. The sections were processed in xylene to remove paraffin and then rehydrate using a series of decreasing ethanol concentrations. Hematoxylin-eosin (HE) staining was performed as follows: hematoxylin staining for 5–10 minutes, differentiation with 1% acid alcohol, and bluing under running water; eosin staining for 1–3 minutes. The sections underwent dehydration in an ethanol series, were cleared using xylene, and mounted with neutral balsam before being examined and photographed under an optical microscope.

### Immunohistochemical staining and evaluation criteria

Endometrial tissue sections were subjected to antigen heat retrieval in citrate sodium buffer (pH 6.0) by microwave heating until boiling and then maintained for 10 minutes, followed by natural cooling to room temperature. After cooling, the sections were rinsed with PBS and incubated with 5% goat serum for 30 minutes at room temperature for blocking. Following serum removal, the sections were incubated with appropriately diluted primary antibodies against BGN (1:500), COL6A3 (1:500), LAMA4 (1:500), and THBS2 (1:500) (Beijing Biosynthesis Biotechnology Co., Ltd., Cat: bs-47363P, bs-100781P, bs-104007P, bs-101297P) overnight at 4 C in a humidified chamber. Following rewarming to room temperature, the sections were washed with PBS and then incubated for 60 minutes with species-matched HRP-conjugated secondary antibodies. After another round of rinsing, color development was performed using a DAB substrate kit. The sections were then washed under running water, lightly counterstained with hematoxylin for nuclear visualization, dehydrated through a graded ethanol series, cleared in xylene, and finally mounted with neutral resin. IHC quantification involved two steps: first, staining intensity was scored from 0 to 3 (0, negative; 1, weak; 2, moderate; 3, strong); second, this score was multiplied by the percentage of positive cells (0–100%), yielding a final score of 0–300. For statistical analysis, the average score was calculated from five randomly selected high-power fields (400× magnification) per section.

### Western blot

Endometrial tissue samples (100 mg) were homogenized on ice using RIPA lysis buffer (1% Triton X-100, 0.1% SDS, 0.5% sodium deoxycholate, 150 mM NaCl, 50 mM Tris-HCl, pH 7.5). The homogenate was cleared by centrifugation at 14,000 rpm for 5min at 4 C, and the protein concentration in the supernatant was determined using a BCA assay kit (#P0012, Beyotime Biotechnology, China). Protein samples (25 μg total protein per sample) were mixed with loading buffer, denatured by heating, and separated by SDS-PAGE. The separated proteins were transferred onto PVDF membranes, blocked with 5% non-fat milk, and then incubated overnight at 4 C with the following primary antibodies: BGN (1:800) (#AF6303, Beyotime Biotechnology, China), COL6A3 (1:800) (#AB231025, Abcam Trading (Shanghai) Co., Ltd.), LAMA4 (1:800) (#AB242198, Abcam Trading (Shanghai) Co., Ltd.), THBS2 (1:800) (#AB89805, Abcam Trading (Shanghai) Co., Ltd.), and GAPDH (1:1000) (#AB181602, Abcam Trading (Shanghai) Co., Ltd.). Subsequently, the membranes were washed three times for 10min each with PBST, followed by incubation with the appropriate horseradish peroxidase (HRP)-conjugated secondary antibody (anti-rabbit IgG, #A0208, Beyotime Biotechnology, China, used at 1:1000 in 5% BSA/PBST) for 1 hour at room temperature. After three additional 10−min washes with PBST, the immunoreactive signals were visualized by coloring the bands using the ECL chemiluminescence method, and the gray values of the bands were quantified using ImageJ software, with GAPDH used as a loading control, and the experiment was performed with at least three independent biological and technical replicates.

### Quantitative real-time PCR

Total RNA (912 ng/μL) was extracted from three tissue samples per group using the RNA Easy Fast Tissue/Cell Kit (TIANGEN; DP451). Reverse transcription was performed using 7 μg of total RNA, and the resulting cDNA was diluted to 700 ng/μL. qPCR reactions were prepared following the manufacturer’s instructions for the 2× SYBR Green qPCR Mix, using cDNA and the gene-specific primers listed in **Table 1**. The amplification protocol comprised an initial denaturation at 95 C for 3min, followed by 40 cycles of denaturation at 95 C for 5 s and annealing/extension at 60 C for 30 s. Relative mRNA expression of BGN, COL6A3, LAMA4, and THBS2 was normalized to ACTB and calculated using the 2–ΔΔCt method.

**Table 1:** Primer sequences.

Gene name	Forward primer	Reverse primer
ACTB	CCTCTATGCCAACACAGTGC	GCTAGGAGCCAGAGCAGTAA
COL6A3	CAGAACCATTGTTTCTCACT	AGGACTACACATCTTTTCAC
BGN	TGTGGCTACTCACCTTGCTG	AGGACACATGGCACTGAAGG
LAMA4	CAGTTTGTCCTCTACCTCGGAAG	CTCACAGGCTTGGAATCCAGGA
THBS2	CTGGCATCGCTGTAGGTTTC	CCTGCTTCCACATCACCAC

### Statistics and analysis

Statistical analysis was performed using Prism 10.1.2. Continuous data are presented as mean ± SD, and group comparisons were conducted using t tests for normally distributed data, with statistical significance set at P < 0.05. For immune cell infiltration comparisons between endometriosis and control groups, Benjamini-Hochberg false discovery rate (FDR) correction was applied to account for multiple comparisons across the 22 immune cell types. An FDR-adjusted p-value < 0.05 was considered statistically significant for these analyses. For correlation analyses between hub genes and immune cells (22 immune cell types × 4 hub genes = 88 comparisons), unadjusted p-values are reported with a stringent threshold of p < 0.01 due to the exploratory nature of these hypothesis-generating analyses. Definition of biological replicates: In this study, n = 6 refers to six animals per group, with each animal considered an independent biological replicate. Tissue samples were collected from ectopic lesions of each animal. For qPCR and Western blot, three technical replicates were performed per biological sample, and the average value was used for statistical analysis.

## Results

### Identification of endometriosis-associated differentially expressed genes

**Figure 2:**
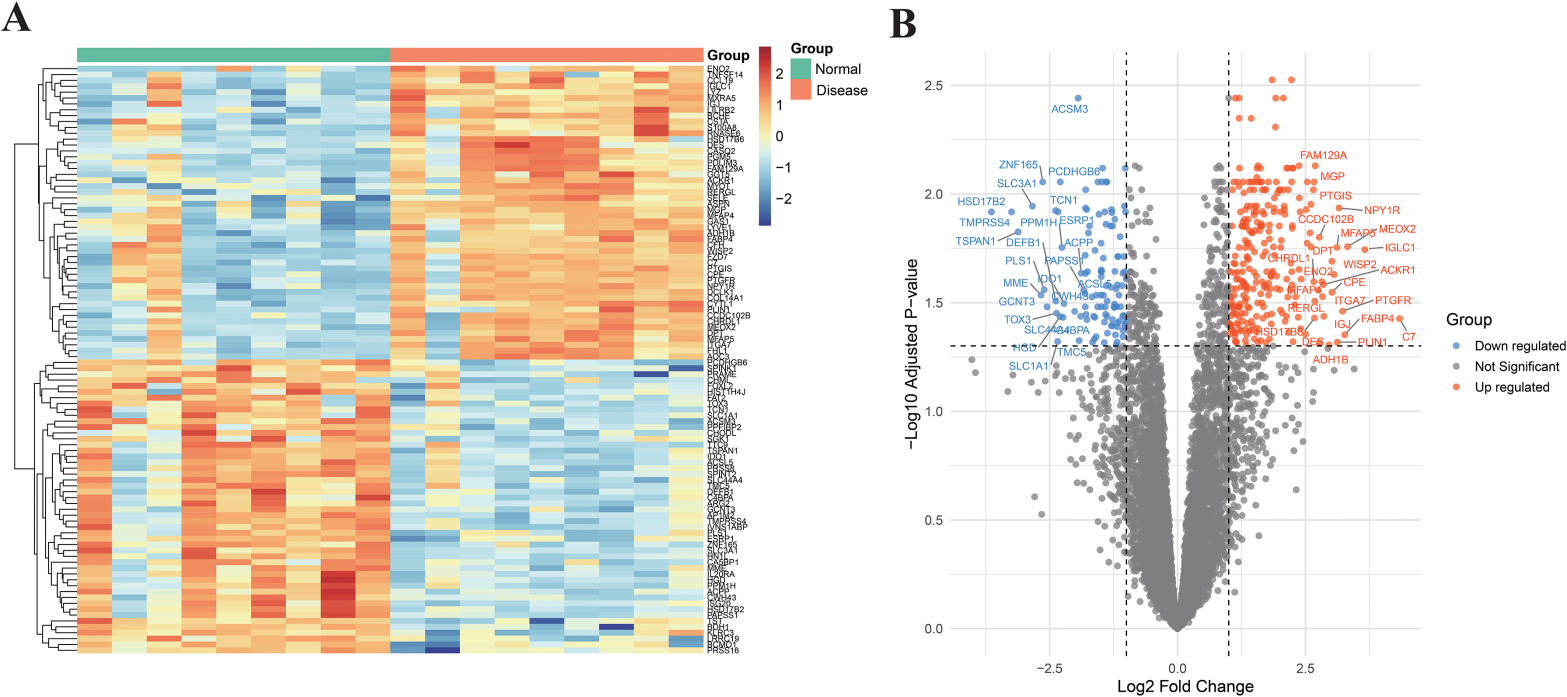
Identification of DEG in endometriosis compared to controls in a training cohort (n=18; 9 endometriosis vs. 9 non-endometriosis controls). **(A)** Volcano plot illustrating the distribution of DEGs. **(B)** Clustered heatmap of DEGs distinguishing the endometriosis group from the control group.

Using a threshold of |log2FC| > 1 and FDR < 0.05, a total of 376 genes were identified as differentially expressed between the endometriosis and control groups in the EM related dataset GSE11691 (9 cases of EM and 9 controls). Among these, 264 genes were significantly upregulated and 112 genes were significantly downregulated in endometriosis compared to controls (**Figure 2**). The top 10 upregulated genes were C7, IGLC1, MEOX2, FABP4, PTGFR, NPY1R, PLIN1, MFAP5, WISP2, and CPE. The top 10 downregulated genes were DEFB1, TCN1, GCNT3, PLS1, ZNF165, MME, SLC3A1, TSPAN1, TMPRSS4, and HSD17B2.

### WGCNA identifies endometriosis-associated gene modules from DEGs

**Figure 3:**
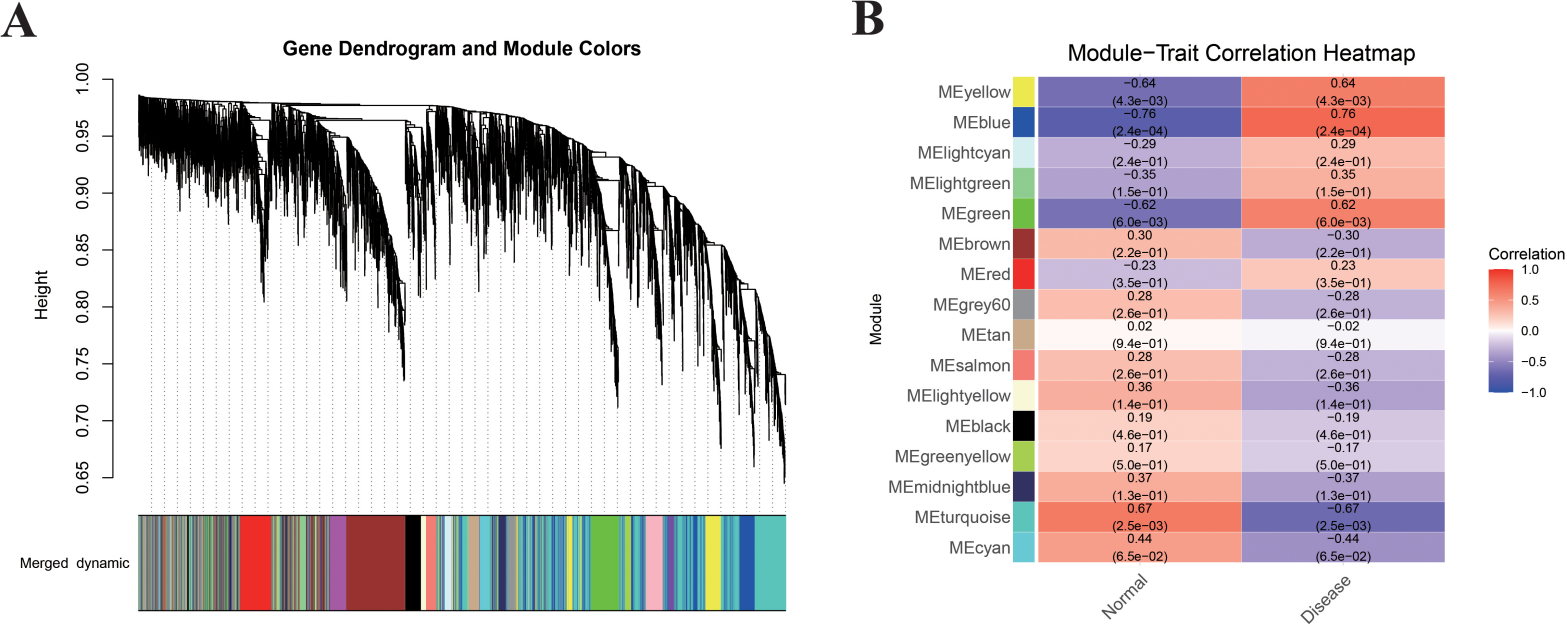
Differential gene expression and WGCNA analysis in endometriosis. **(A)** Soft-thresholding power selection for WGCNA construction. **(B)** Module-trait correlations between identified co-expression modules and clinical traits, presented as correlation coefficients with corresponding p-values.

While differential expression analysis identifies individual genes that change between conditions, it does not capture coordinated transcriptional programs. To identify co-expressed gene modules associated with endometriosis, we performed WGCNA. As shown in **Figure 3A**, a soft-thresholding power of β = 8 was selected to achieve a scale-free network topology (R² = 0.90). Using this threshold, we identified several co-expression modules. Among these, the MEblue, MEyellow, and MEgreen modules showed substantial positive correlation with endometriosis (R > 0.5, p < 0.05 for all; **Figure 3B**). Additionally, there was a significant difference (p < 0.05) in the distribution of gene significance scores across modules, indicating that some modules were more biologically relevant to the disease than others. To further validate the relationship between module membership and disease relevance, we performed an intra-module analysis for the MEblue module, which revealed a strong positive association between module membership (MM) and gene significance (GS) (R=0.76, p < 0.05; **Figure 3B**). This finding indicates that genes more central to the MEblue module tend to be more strongly associated with endometriosis. Therefore, the 1,071 genes in the MEblue module were selected as endometriosis-associated gene candidates for subsequent analyses.

### Integrated analysis identifies biologically enriched pathways in endometriosis

**Figure 4:**
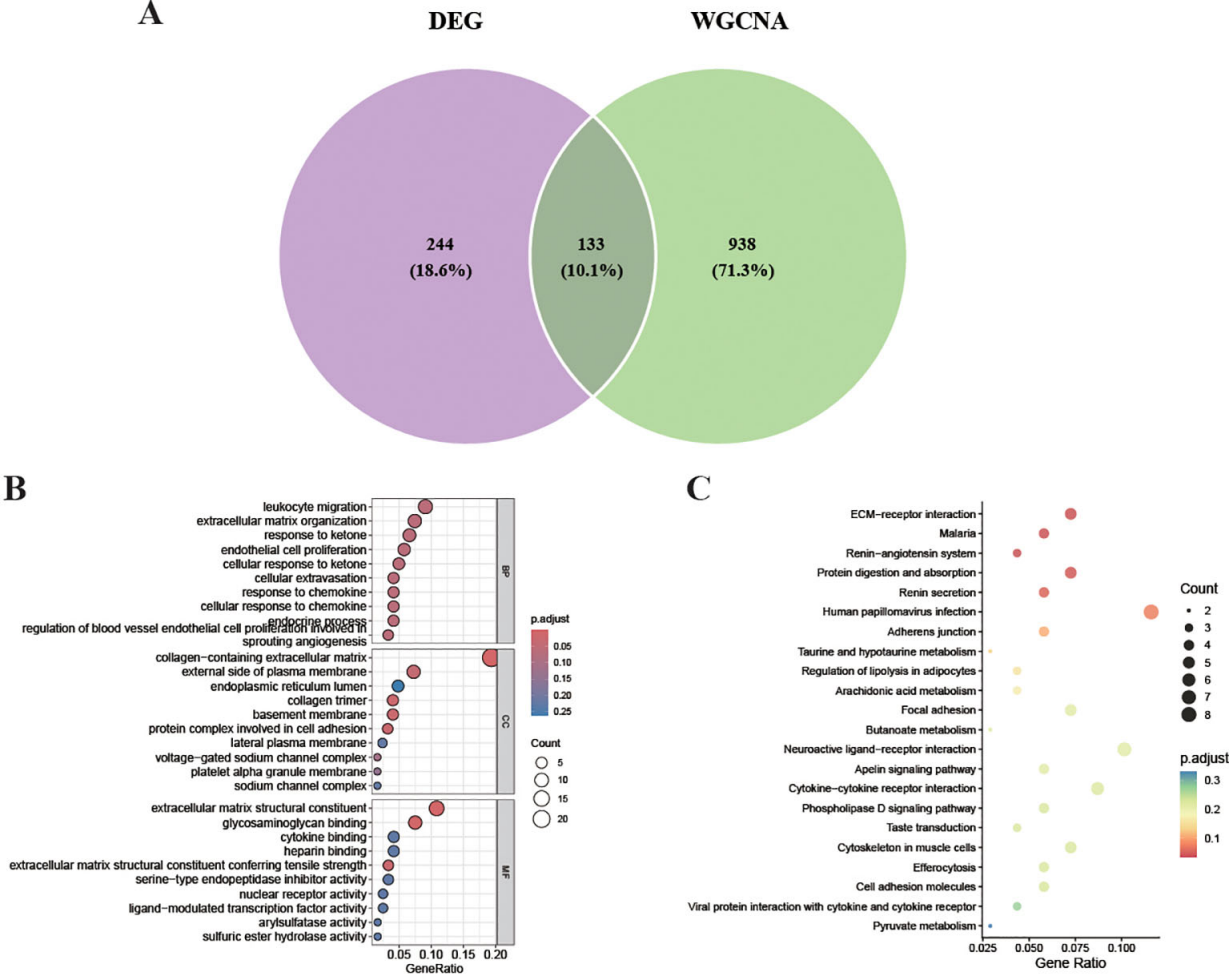
Identification and functional enrichment of genes shared between DEGs and co−expression modules. **(A)** a Venn diagram showing the overlap between the DEGs and the MEblue WGCNA module, along with the corresponding. **(B)** GO and **(C)** KEGG enrichment analyses of these overlapping genes.

To identify genes that are both individually differentially expressed and co-expressed in disease-relevant modules, we intersected the DEG set (376 genes) with the MEblue module genes (1,071 genes). This intersection yielded 133 overlapping genes (**Figure 4A**), representing high-confidence endometriosis-associated candidates. To elucidate the biological functions and pathways of these 133 genes, we performed Gene Ontology (GO) and Kyoto Encyclopedia of Genes and Genomes (KEGG) enrichment analyses. The GO analysis revealed that these genes were strongly associated with extracellular matrix (ECM) related terms, including leukocyte movement, extracellular matrix structural constituent, and collagen-containing extracellular matrix (**Figure 4B**). The KEGG pathway analysis showed significant enrichment in ECM-receptor interaction, malaria, the renin-angiotensin system, protein digestion and absorption, and human papillomavirus infection (**Figure 4C**). These results suggest that ECM remodeling and cell adhesion pathways play central roles in endometriosis pathogenesis.

### Identification of seven characteristic endometriosis genes through integrated PPI network and machine learning analysis

**Figure 5:**
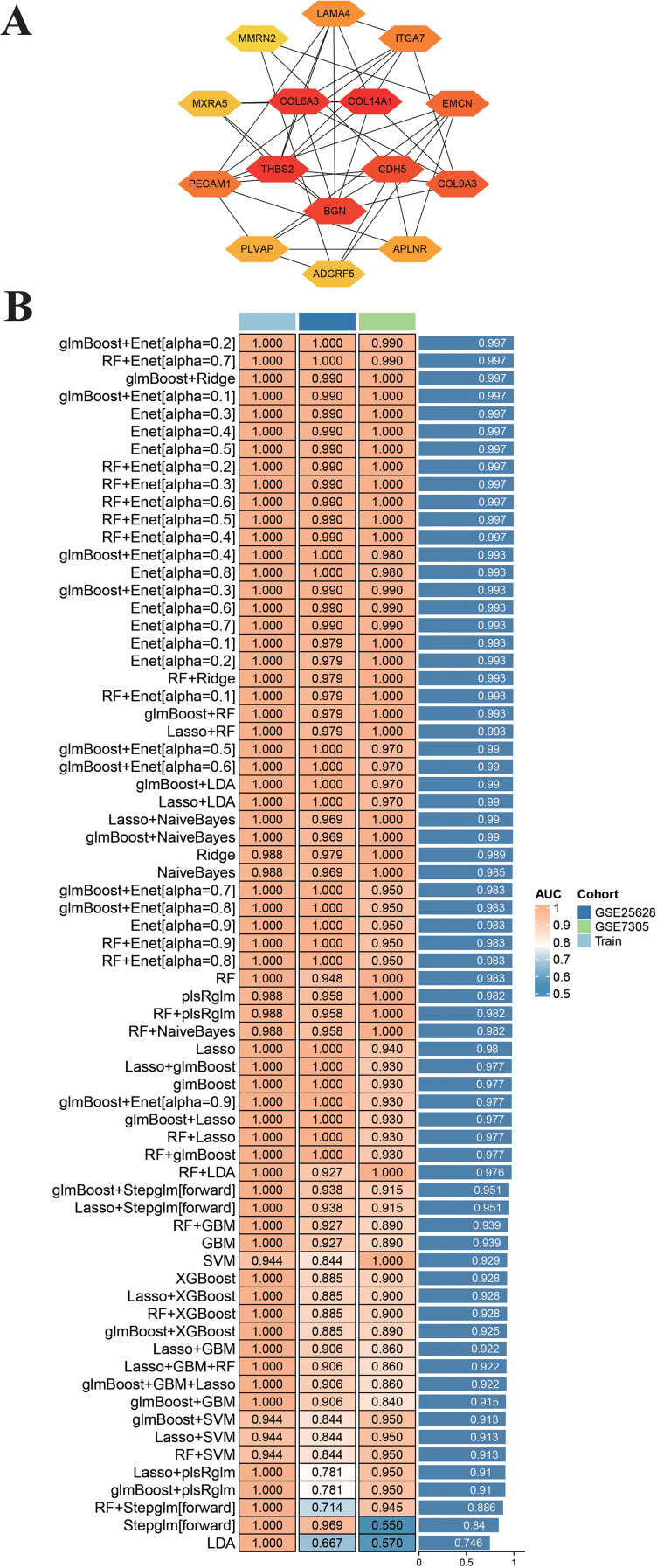
**(A)** The top 15 pivotal genes in the PPI network. **(B)** 113 machine learning algorithm combinations evaluated via 10-fold cross-validation.

Based on an integrative bioinformatics approach combining PPI network analysis and machine learning, we identified characteristic genes for endometriosis. The initial PPI network was constructed, and the Degree algorithm in cytoHubba was utilized to pinpoint the 15 most interconnected hub genes (**Figure 5A**). To refine this list into a robust diagnostic signature, we performed a machine learning-based integration of the expression profiles of these 15 candidate genes. A comprehensive comparative analysis was conducted by constructing and evaluating a total of 113 distinct predictive classification models. The batch effects between the GSE25628 and GSE7305 datasets were removed using the R package sva, and the effectiveness of this correction was evaluated by distribution boxplots (**Supplementary Figures 1A, B**) and PCA plots (**Supplementary Figures 1C, D**). Both the boxplot and PCA results demonstrated that batch effects in the combined psoriasis dataset were effectively eliminated. These models were applied to our training dataset and validated on independent cohorts (GSE25628 and GSE7305) using a cross-validation framework to ensure generalizability. The performance of these algorithms was rigorously assessed, with 12, such as GLMBoost +Enet and RF+Ridge, demonstrating high AUC scores above 0.997 in the training phase. The GLMBoost + Enet algorithm was employed for final feature selection. This algorithm combines gradient boosting with elastic net regularization, which performs embedded feature selection by shrinking the coefficients of irrelevant or redundant genes toward zero. Only genes with non-zero coefficients in the optimized model were retained as predictive features. Applying this algorithm to the 15 PPI-derived hub genes yielded a refined set of seven genes (COL14A1, COL6A3, THBS2, BGN, COL9A3, LAMA4, APLNR) that together achieved the highest cross-validated predictive performance (AUC > 0.997). These seven genes were therefore considered the most informative signature for distinguishing endometriosis from controls. This signature represents a refined panel of candidate tissue markers, derived from a robust computational pipeline, offering strong potential for elucidating the pathogenesis and improving the diagnosis of the condition.

**Figure 6:**
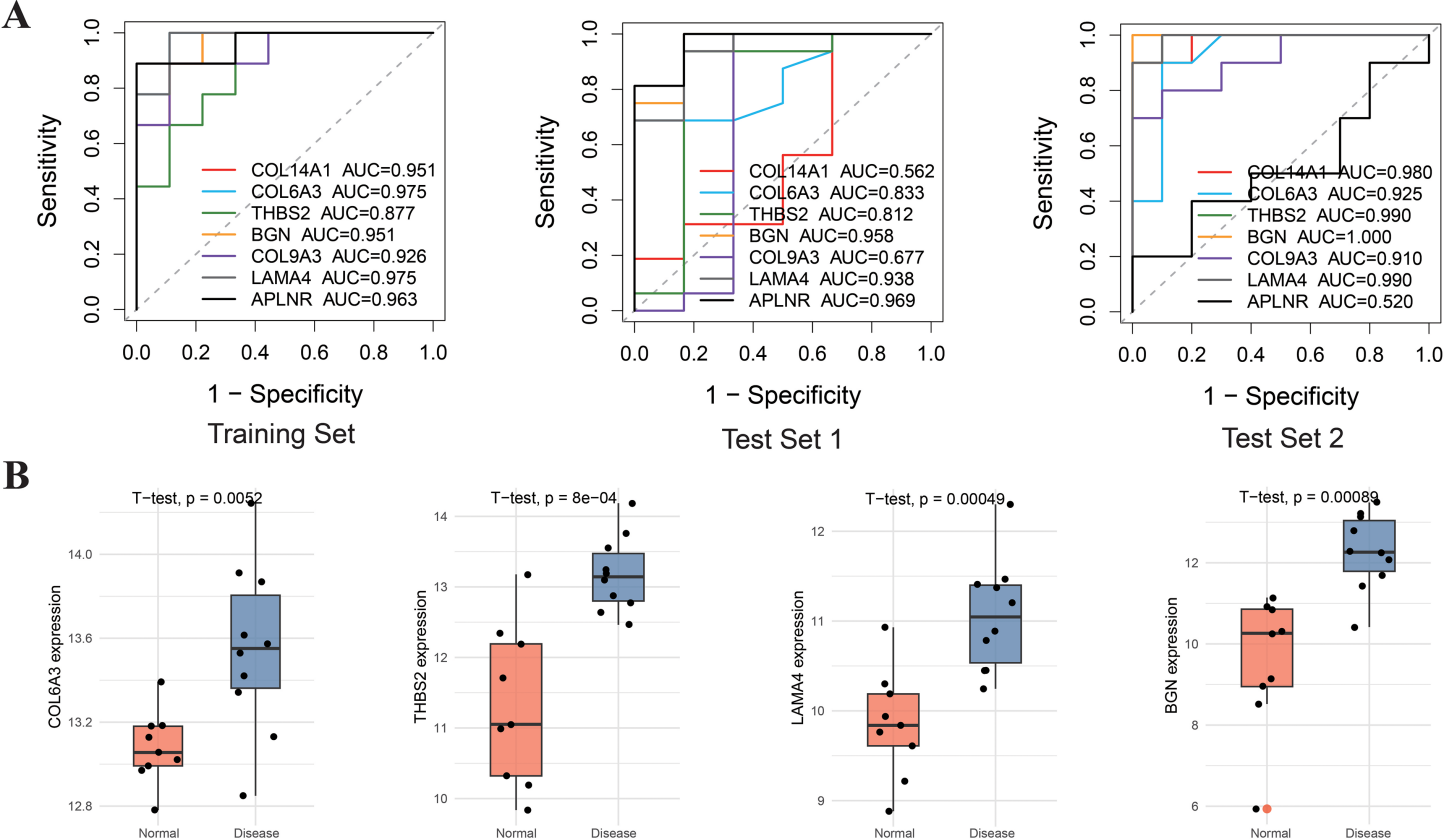
ROC curve and expression level of characteristic genes. **(A)** ROC curves of the four characteristic genes are presented for the training set (GSE11691, n=18), test set 1 (GSE25628, n=14), and test set 2 (GSE7305, n=20) **(B)** Expression levels of four hub genes in external validation sets.

To assess the diagnostic value of the seven signature genes, ROC analysis was conducted across both the training and testing cohorts. The results demonstrated that COL14A1, COL6A3, BGN, and LAMA4 achieved AUC values exceeding 0.95 in the training cohort (**Figure 6A**), indicating high sensitivity and specificity in distinguishing endometriosis from controls within the training set. The diagnostic relevance of THBS2 was also notable. Moreover, a diagnostic model combining two core genes was developed, showing notably better performance in both the training and testing groups. Two independent datasets (GSE25628 and GSE7305 as test sets) were used to further validate the diagnostic accuracy, clinical relevance, and expression patterns of COL6A3, BGN, LAMA4, and THBS2. Although the datasets showed differences in sample origins, the diagnostic potential and increased expression of these genes were consistently confirmed. Specifically, COL6A3 (all AUC > 0.80), BGN (all AUC > 0.95), LAMA4 (all AUC > 0.90), and THBS2 (all AUC > 0.80) maintained strong discriminatory power. External validation cohorts also revealed a significant upregulation pattern of four core genes in endometriosis samples (p < 0.001) (**Figure 6B**). Based on these findings, COL6A3, BGN, LAMA4, and THBS2 were ultimately identified as key target genes in endometriosis.

### Analysis of immune infiltration

**Figure 7:**
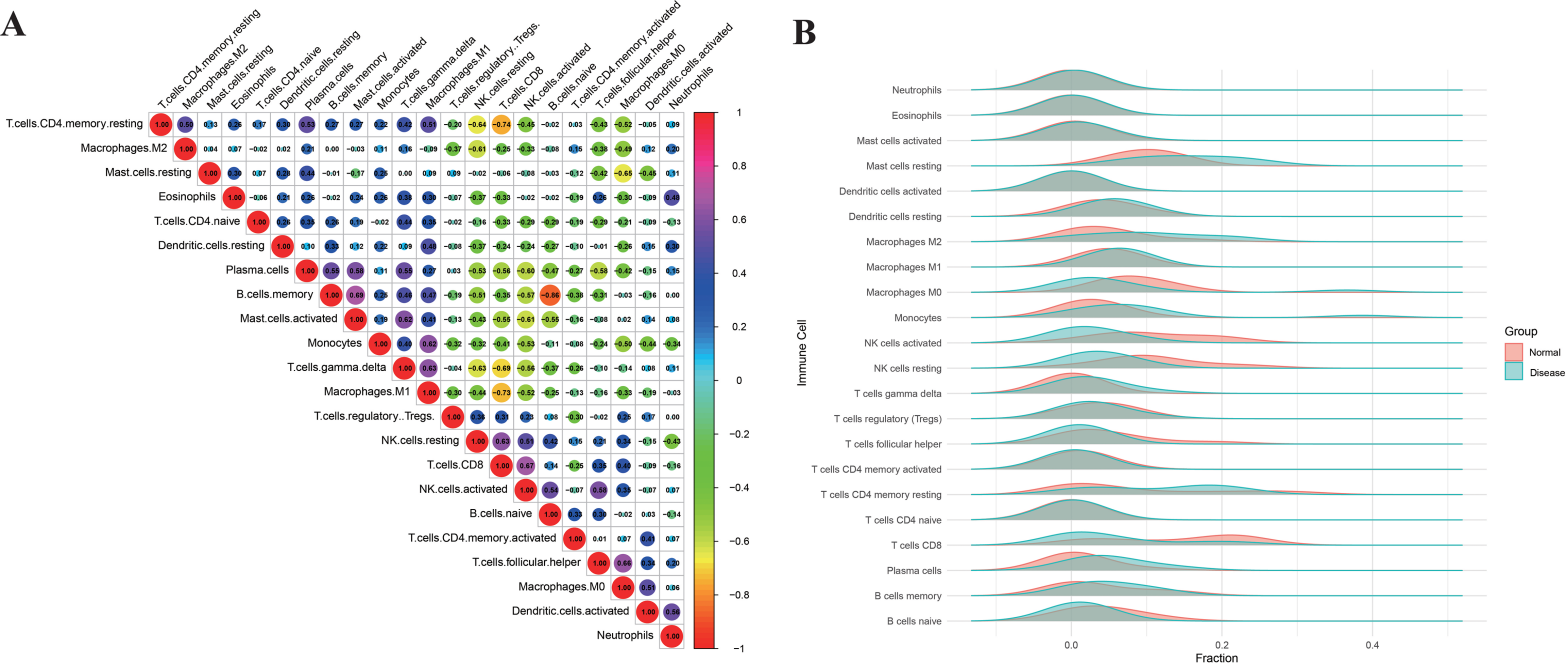
Intercorrelation analysis of key immune cell types.

Correlation analysis revealed modular interactions among immune subsets in the endometriosis microenvironment (**Figure 7A**). An inhibitory myeloid cluster (M2 macrophages, resting dendritic cells, and resting mast cells) exhibited positive internal correlations (r = 0.4–0.7). This cluster inversely correlated with effector immune cells, including CD8^+^ T cells (r ≈ –0.64) and activated NK cells (r ≈ –0.61), while M1 and M2 macrophages showed antagonism (r ≈ –0.74). Within the effector immune module, activated NK cells positively correlated with CD8^+^ T cells (r ≈ 0.63), and memory B cells closely associated with plasma cells (r ≈ 0.86). Naïve CD4^+^ T cells and γδ T cells showed weak correlations with most subsets, indicating functional independence. Comparative immune profiling revealed distinct immune cell distributions between control and endometriosis groups (**Figure 7B**). The disease group showed elevated memory B cells, follicular helper T cells, CD8^+^ T cells, and an increased activated-to-resting NK cell ratio, alongside a shift toward M1-dominant macrophage polarization.

### Altered immune cell infiltration and hub gene associations in endometriosis cohorts

**Figure 8:**
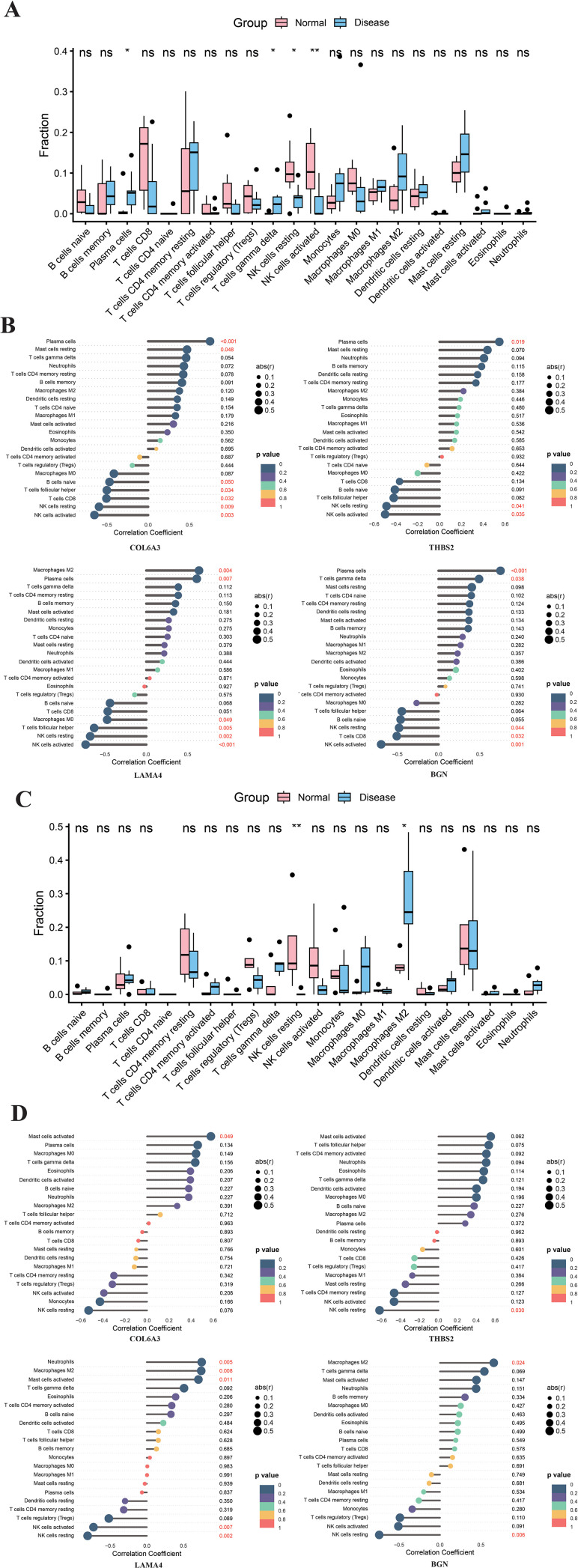
Exploring immune characteristics in endometriosis and immune infiltration associated with core genes. **(A)** Profiles of 22 key immune cell types in the test set (GSE25628 + GSE7305, n = 34; left: control, right: endometriosis). **(B)** Stacked bar plots for the external validation set (GSE23339, n = 19). **(C)** Lollipop chart illustrating correlations between core genes and infiltrating immune cells in the training set. **(D)** Lollipop chart for the validation set. Only correlations with p < 0.01 (unadjusted) are shown. Red indicates positive correlation, blue indicates negative correlation. **p < 0.01; *p < 0.05.

In both the training and validation cohorts of endometriosis, plasma cells and gamma delta T cells displayed significantly elevated infiltration levels and were positively correlated with the central hub genes. Conversely, distinct patterns of reduced infiltration were observed for other immune subsets. In the training cohort, both resting and activated NK cells showed markedly diminished infiltration and a negative association with the hub genes. Similarly, within the validation cohort, resting NK cells and M2 macrophages exhibited significantly decreased infiltration levels alongside negative correlations with the central genes (**Figure 8**). These consistent findings across cohorts, particularly regarding NK cells, suggest a pivotal role for this immune subset in the hub gene-regulated immune pathways implicated in endometriosis.

### Histopathological and gene expression alterations in endometriosis model

**Figure 9:**
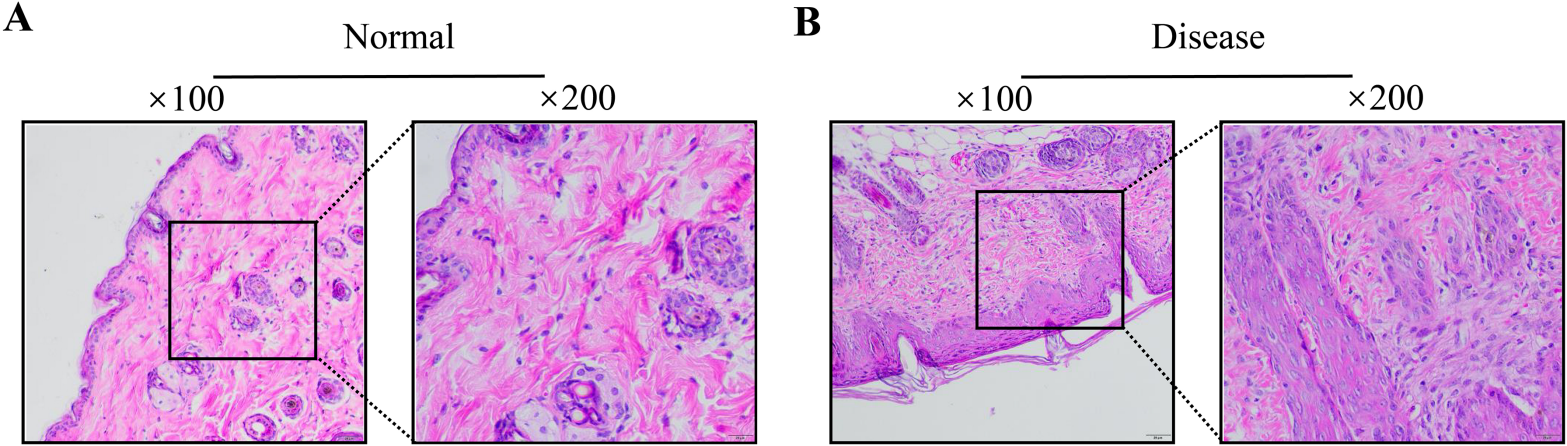
HE staining results of uterine tissue from mice in each group (100×, 200×). (n = 6 per group). **(A)** Control group, **(B)** Pathological group.

**Figure 10:**
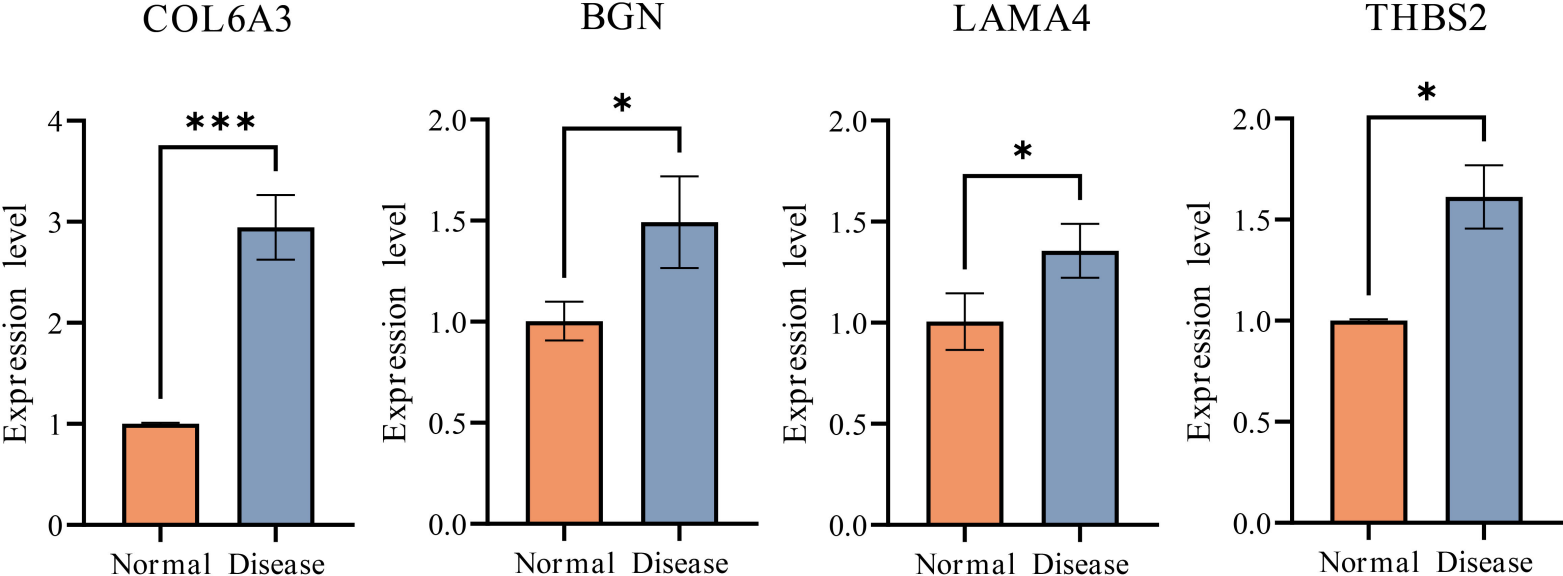
Normalized expression levels of disease-associated genes (COL6A3, BGN, LAMA4, and THBS2) across comparative groups in mice endometrial tissues (n = 6 per group). ***p < 0.001; *p < 0.05.

Histopathological examination of mice uterine tissue revealed characteristic morphological changes between groups. In the control group, light microscopy showed normal endometrial architecture with abundant glandular epithelium, thickened lamina propria, increased stromal cellularity, and orderly glandular arrangement without pathological alterations. The endometriosis model group showed clear changes in the ectopic endometrium, with significant thickening, decreased glandular density, and widespread infiltration of inflammatory cells, reflecting notable alterations in tissue structure, cell makeup, and local inflammatory conditions. These findings confirm the successful establishment of the endometriosis animal model (**Figure 9**). In line with pathological findings, qPCR analysis showed a marked increase in the expression of extracellular matrix-related genes in the disease group when compared to the normal group. Specifically, the median expression levels of COL6A3 increased from 0.95 to 2.40, while BGN, LAMA4, and THBS2 each increased from 0.95 to 1.35. Each gene showed a consistently higher expression range in the disease group, which further supports the association between endometriosis and the upregulated transcription of key extracellular matrix components (**Figure 10**).

### Expression levels of COL6A3, BGN, LAMA4, and THBS2 proteins in ectopic endometrium

**Figure 11:**
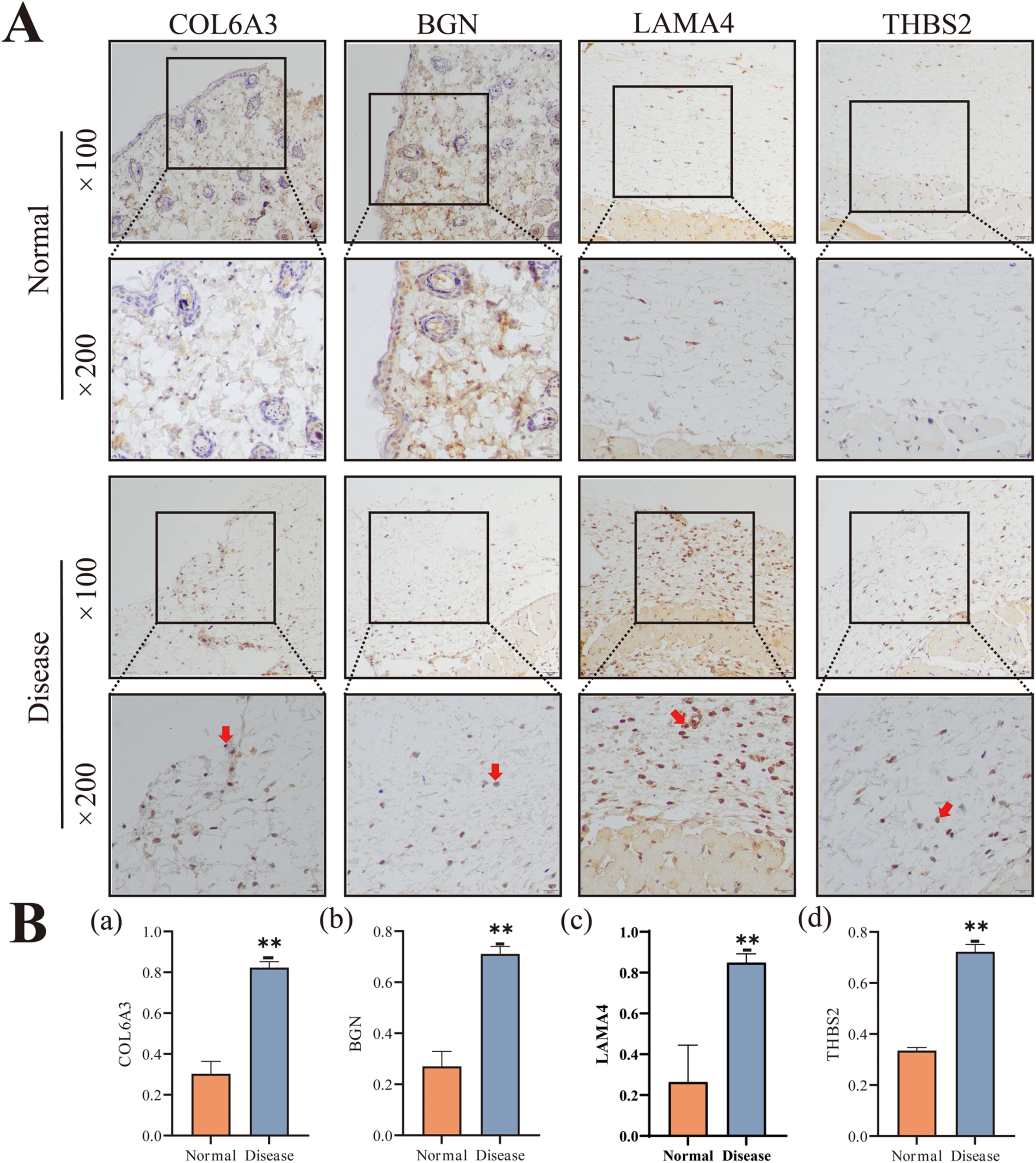
**(A)** Positive protein expression of COL6A3, BGN, LAMA4, and THBS2 in the ectopic endometrium of mice from each group (immunohistochemical staining) (n = 6 per group). **(B)** Histogram showing positive protein expression levels of COL6A3, BGN, LAMA4, and THBS2 in the ectopic endometrium of mice from each group (a) COL6A3 (b) BGN (c) LAMA4 (d) THBS2.

**Figure 12:**
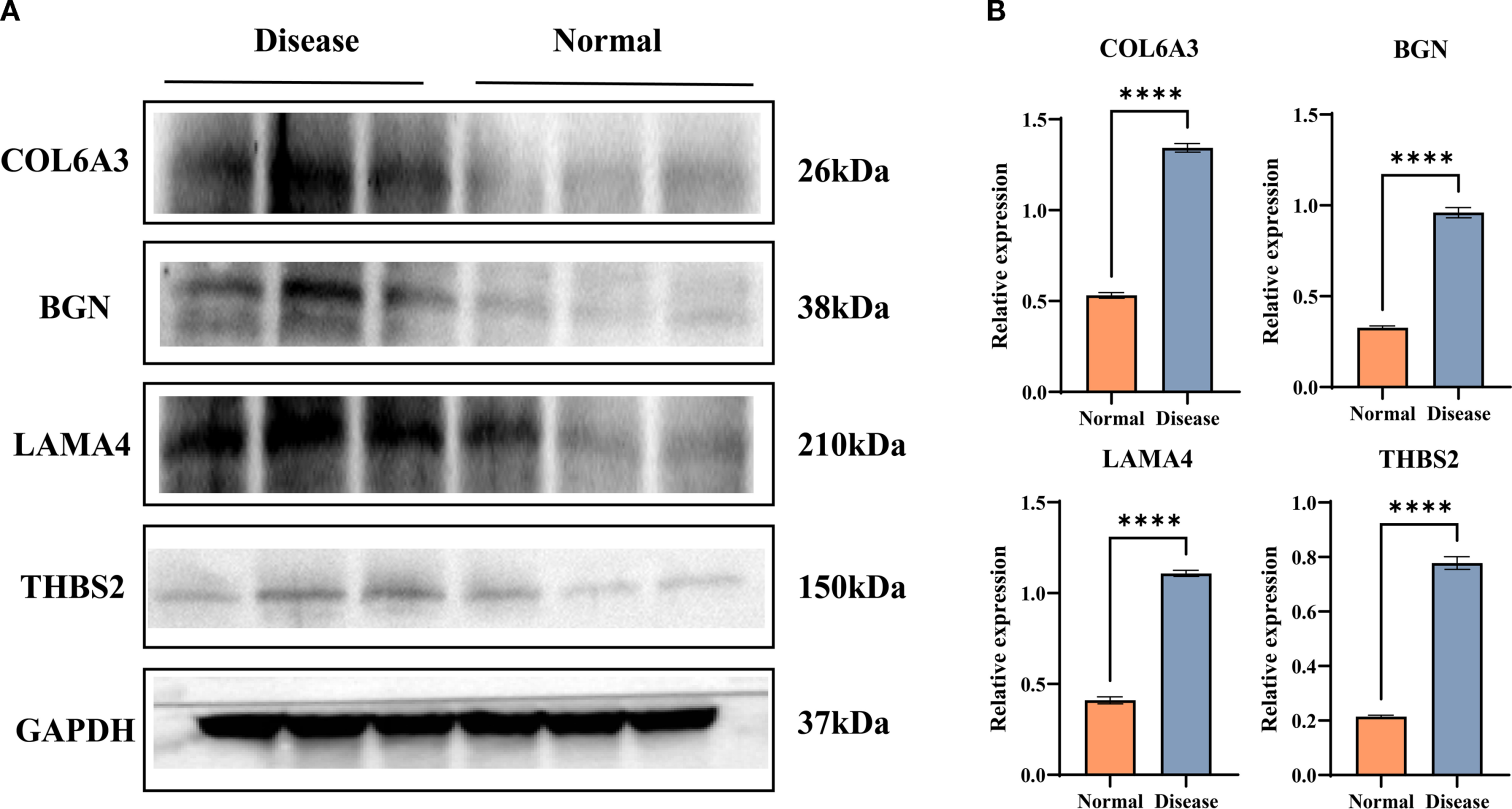
Western blot analysis of COL6A3, BGN, LAMA4, and THBS2 protein expression in mice endometrial tissues. (n = 6 per group). **(A)** Representative strips of western blotting results. **(B)** Quantitative analysis of protein expression levels: (a) COL6A3, (b) BGN, (c) LAMA4, and (d) THBS2. Data are presented as mean ± SD; **** *P*<0.0001, *P*<0.0001 versus the normal group.

To investigate the roles of COL6A3, BGN, LAMA4, and THBS2 in the pathogenesis of endometriosis, we first examined the histological features of endometrial tissues from different groups. As shown in **Figure 11**, mice in the disease group exhibited marked inflammatory cell infiltration in the endometrial tissue, accompanied by increased expression levels of all four proteins. We further validated the protein expression changes using Western blot analysis. Relative to controls, the model group showed significantly elevated protein expression levels of COL6A3, BGN, LAMA4, and THBS2 (**Figure 12**). These results indicate that abnormal expression of COL6A3, BGN, LAMA4, and THBS2 may play a key role in the progression of endometriosis.

## Discussion

The research has achieved groundbreaking advancements in merging diverse methodologies and examining immune regulatory systems. Initially, we amalgamated bioinformatics and machine learning’s systematic validation approach to methodically ascertain the significance of COL6A3, BGN, LAMA4, and THBS2 in endometriosis through a multi-omics lens. These genes were consistently upregulated in endometriosis tissues across multiple datasets, demonstrated high diagnostic accuracy (AUC > 0.80), and showed significant correlations with immune cell infiltration, particularly resting NK cells and M1 macrophages. Experimental validation in a mice endometriosis model confirmed their upregulation at both mRNA and protein levels. Below we discuss the potential roles of these genes in endometriosis pathogenesis and their implications for diagnosis and therapy.

This study’s methodological framework offers distinct benefits in terms of both singular and comprehensive analysis. In terms of singular methodologies, the research minimized the unpredictability in analyzing individual genes via modular bioinformatics screening (WGCNA/PPI), enhanced the precision of diagnoses through machine learning cross-validation (RF + NaiveBayes), and determined the causal link between genes and diseases by employing genetic diversity to prevent environmental misunderstandings. In the realm of integration, our advancement from correlational studies to analyzing mechanisms was realized by creating and confirming multi-omics evidence sequences from biomarkers, examining immune interactions to genetic causes, and integrating HEIDI tests to remove linkage disruptions. Employing a multi-omics approach successfully circumvents the constraints of a solitary method, offering a novel biological viewpoint on the development of endometriosis. Given the likely lack of recognition for many endometriosis cases, prompt diagnosis and treatment are vital (24, 25). Diagnostic laparoscopy with histological confirmation has traditionally been considered the gold standard for endometriosis diagnosis; however, significant diagnostic delays in the clinical setting still prevail, despite the current reliance on endoscopic and histological evaluation (26). Hence, identifying biomarkers in endometriosis patients is imperative. This research pursues the discovery of potential tissue-based candidate markers and investigates their interconnections and diagnostic utility.

Collagen type VI alpha 3 (COL6A3), a component of extracellular matrix fibrils, has been implicated in oncogenesis, with its overexpression correlated specifically with high-grade ovarian carcinoma. This finding aligns with the established epidemiological link between endometriosis and ovarian cancer—women with endometriosis have a 4.2-fold higher risk of ovarian cancer, rising to 9.7-fold for deep infiltrating disease (27). Emerging evidence suggests COL6A3 may mechanistically connect these two conditions, as its overexpression in endometriosis tissues correlates with worse survival, and targeting COL6A3 suppresses ovarian cancer cell aggressiveness in preclinical models (28). Thus, COL6A3 may serve not only as a diagnostic marker but also as a potential therapeutic target to slow malignant transformation. In the present study, both mRNA and protein expression levels of COL6A3 were significantly elevated in the endometriosis model mouse. Combined with the histopathological observations of endometrial thickening and inflammatory cell infiltration, these results suggest that high COL6A3 expression may promote invasive growth and tissue disorganization in ectopic lesions. This finding provides preliminary experimental evidence supporting COL6A3 as a potential biomarker associated with stromal remodeling in endometriosis.

Biglycan (BGN), a member of the small leucine-rich proteoglycan (SLRP) family, is a multifunctional extracellular matrix component critically involved in regulating diverse cellular processes. These include, but are not limited to, cell adhesion, migration, proliferation, autophagy, inflammation, and the modulation of cell morphology and motility (29–32). Its oncogenic potential is highlighted in the context of endometrial cancer, where BGN has been demonstrated to enhance cancer cell migration and invasion (10). Relevant to endometriosis, bioinformatic analyses have consistently identified BGN as a significant common gene linking endometriosis and endometrial carcinoma (11, 33), suggesting a shared pathogenic mechanism. This computational prediction has been substantiated by experimental evidence. Immunohistochemical studies confirm the upregulation of BGN protein in endometriotic lesions. Furthermore, this elevated expression is associated with pathways involved in estrogen metabolism and action, pointing to a potential mechanistic role for BGN in the estrogen-dependent growth and survival of endometriotic tissue. In summary, BGN emerges as a compelling molecule in endometriosis research. Its established pro-migratory and pro-invasive functions in a related gynecological cancer, coupled with its bioinformatic prioritization and validated overexpression linked to a core hormonal pathway in endometriosis, position it as a promising candidate for further investigation into the progression and maintenance of endometriotic implants. Notably, in the present study, the histological finding of prominent inflammatory cell infiltration, together with BGN upregulation, suggests that abnormal BGN expression may serve as a molecular indicator of endometriosis-associated stromal inflammatory responses.

LAMA4 expression was markedly elevated in endometriosis compared with normal tissues. The underlying mechanisms of this increase are complex. As a crucial member of the laminin family, LAMA4 contributes substantially to extracellular matrix (ECM) stability (34). Enhanced expression may promote ECM densification and rigidity, forming a physical barrier that impedes cytotoxic T−cell infiltration into lesion sites, thereby facilitating immune evasion (35). Additionally, through interaction with receptors such as integrins, LAMA4 triggers intracellular signaling cascades involving FAK and PI3K/AKT, which enhance cell survival, proliferation, and apoptotic resistance, thus diminishing immune−mediated cytotoxicity (36). The present study demonstrated that both mRNA and protein expression of LAMA4 were significantly elevated in the model group, indicating that upregulation of LAMA4 may represent a molecular feature of basement membrane dynamic remodeling in endometriosis.

The pathogenesis of endometriosis is often associated with abnormal changes in the structure and composition of the endometrial extracellular matrix. Studies have revealed significant collagen remodeling dysregulation and elevated lysyl oxidase levels in ectopic endometrial tissues, which are considered key factors in disease development. In this process, thrombospondin-2 (THBS2), encoded by the gene *THBS2* (MIM 188061), plays an important role (37). THBS2 is a secreted homotrimeric matricellular protein widely distributed in the extracellular matrix and involved in the regulation of collagen homeostasis through both direct and indirect mechanisms (37, 38). Expression changes of THBS2 correlate with epithelial-mesenchymal transition (EMT) markers (39), suggesting a role in the invasive phenotype of endometriotic lesions. Our KEGG analysis further identified ECM-receptor interaction as a top enriched pathway, reinforcing the importance of ECM remodeling in endometriosis pathogenesis. Consistent with these lines of evidence, the present study found that THBS2 expression was significantly upregulated in the endometriosis model, suggesting that it may participate in the regulation of the stromal microenvironment in ectopic lesions.

Several limitations of this study should be acknowledged. First, while we employed WGCNA on DEGs as an initial screening strategy, we recognize that this approach may bias network topology by excluding genes with subtle expression changes but strong co-expression relationships. Nevertheless, the convergence of evidence from PPI analysis, machine learning, external validation, and experimental verification supports the robustness of our four candidate genes. Second, the diagnostic performance of COL6A3, BGN, LAMA4, and THBS2 was evaluated only in tissue samples (public datasets and mice ectopic endometrium); their utility in non-invasive clinical specimens (e.g., blood, urine) requires future validation. Third, animal experiments were conducted in mice, and findings should be cautiously translated to human endometriosis. Fourth, while we applied strict cross-validation to prevent data leakage, we did not employ nested cross-validation for hyperparameter tuning. However, the use of an independent external validation set (GSE23339) already helps mitigate concerns about serious overfitting. Nested cross-validation remains a consideration for future work to enhance rigor. The fact that our four genes performed consistently well across multiple independent datasets gives us confidence in their robustness.

## Conclusion

In this study, candidate genes associated with endometriosis, such as COL6A3, BGN, LAMA4, and THBS2, were identified through bioinformatics screening and subsequently validated by experiments, suggesting their potential as candidate tissue diagnostic markers. These genes were consistently upregulated in endometriotic tissues across multiple datasets and demonstrated strong discriminatory power in distinguishing patients from controls. These candidate genes are primarily involved in key pathways related to endometriosis, including extracellular matrix remodeling and cell adhesion.

## Data Availability

Publicly available datasets were analyzed in this study. This data can be found here: https://www.ncbi.nlm.nih.gov/geo/.
