# Supplementary figures and images for: Bioinformatics and machine learning-driven discovery of candidate tissue diagnostic markers for endometriosis with experimental verification
Source: Front Endocrinol (Lausanne). 2026 May 22;17:1802649. doi: 10.3389/fendo.2026.1802649 (PMC13236618; doi:10.3389/fendo.2026.1802649)

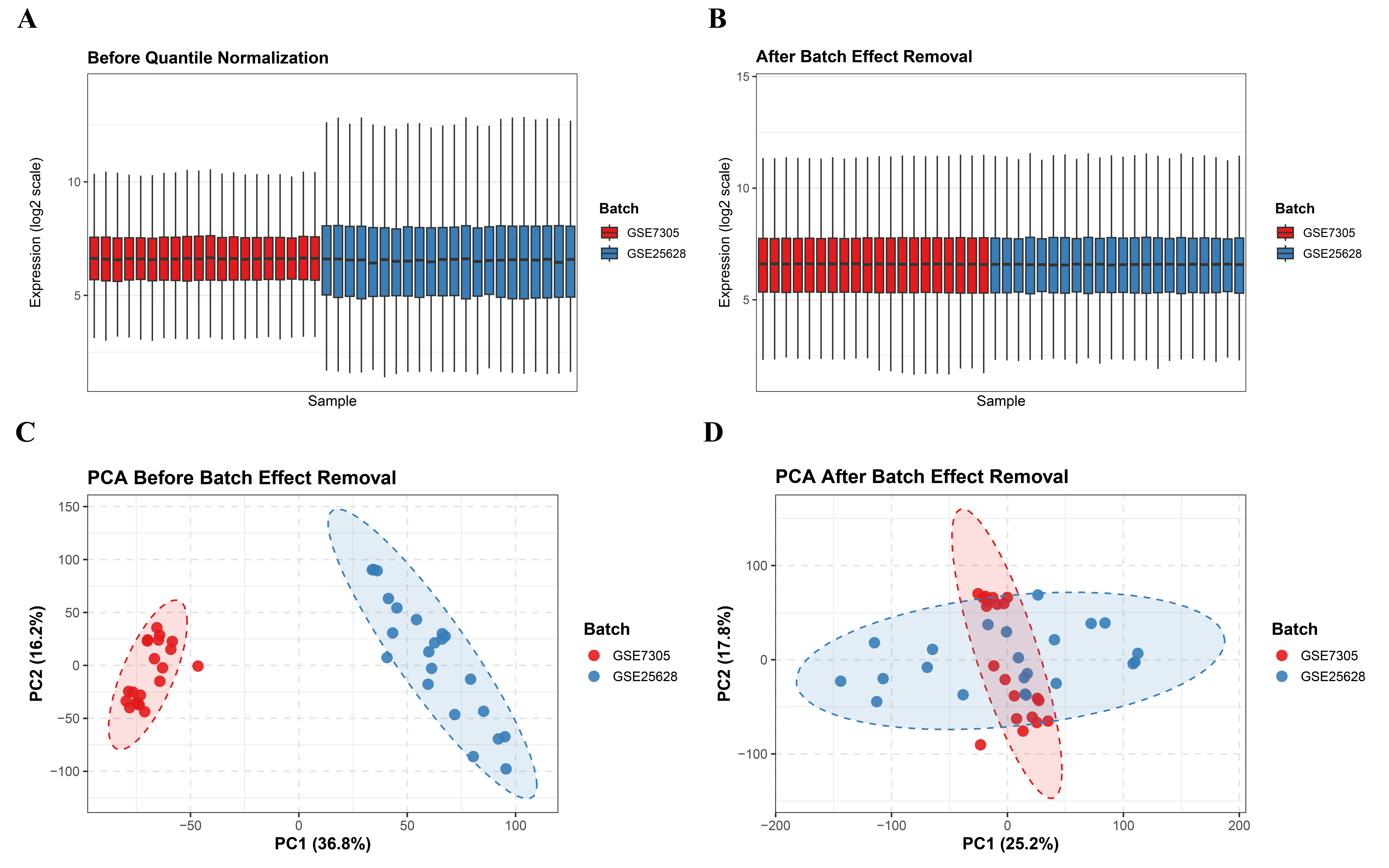

Supplement: Supplementary file 1 [file Image1.jpeg]
